# Intermediate-band dynamics of quantum dots solar cell in concentrator photovoltaic modules

**DOI:** 10.1038/srep04792

**Published:** 2014-04-25

**Authors:** Tomah Sogabe, Yasushi Shoji, Mitsuyoshi Ohba, Katsuhisa Yoshida, Ryo Tamaki, Hwen-Fen Hong, Chih-Hung Wu, Cherng-Tsong Kuo, Stanko Tomić, Yoshitaka Okada

**Affiliations:** 1 Research Center for Advanced Science and Technology, The University of Tokyo, 4-6-1 Komaba, Meguro-ku, Tokyo 153-8904, Japan; 2 Solar Energy Program, Institute of Nuclear Energy Research, Taoyuan, Taiwan; 3 Joule Physics Laboratory, School of Computing Science and Engineering, University of Salford, Manchester M5 4WT, UK; 4 Research Center for Advanced Science and Technology, The University of Tokyo, 4-6-1 Komaba, Meguro-ku, Tokyo 153-8904, Japan

## Abstract

We report for the first time a successful fabrication and operation of an InAs/GaAs quantum dot based *intermediate band solar cell* concentrator photovoltaic (QD-IBSC-CPV) module to the IEC62108 standard with recorded power conversion efficiency of 15.3%. Combining the measured experimental results at Underwriters Laboratory (UL®) licensed testing laboratory with theoretical simulations, we confirmed that the operational characteristics of the QD-IBSC-CPV module are a consequence of the carrier dynamics via the intermediate-band at room temperature.

The intermediate-band solar cell (IBSC) is one of the most promising candidates for the next generation of photovoltaic devices, with a detailed balance efficiency of 63.2% under idealised conditions and maximum concentration^1^. IBSCs require light concentration not only to reduce the device cost but also to ensure optimal operation when carrier photogeneration is outperforming the recombination processes via the intermediate-band (IB). Although the *intermediate-band* term is used exstensively in the literature for this concept of SC device, we would like to point out that those are actually a collection of *intermediate-states* of quantum dots in a particular surface or vertical arrangement, and not necessarily perfectly periodic or infinite. To date, great effort has been devoted to the fabrication of IBSC devices based on inorganic quantum dots and the study of their principle of operation^2^,^3^,^4^,^5^,^6^,^7^,^8^,^9^,^10^,^11^, with limited or no evidence of IB operation. Despite significant advances, evaluation of carrier dynamics via IB for an IBSC in a concentrating photovoltaic (CPV) module configuration presents both fundamental and technical obstacles, limiting its application and possible future commercialisation.

We report here the first successful fabrication of an InAs/GaAs quantum dot based IBSC concentrator photovoltaic (QD-IBSC-CPV) that works in the IBSC regime, with recorded power conversion efficiency of 15.3%. Following experimental results and theoretical simulations, we confirm that the operation characteristics of the QD-IBSC-CPV module are a consequence of carrier dynamics via states in the intermediate-band at room temperature, and not thermal escape.

The concentrating photovoltaic (CPV) module contains solar receivers and concentrating optical systems. Fig. 1 shows a picture of the fabricated CPV module, which contains two separate parts with four series-connected GaAs control sub-modules on one side and four InAs/GaAs QD-IBSC-CPV sub-modules on the other. The InAs/GaAs QD-IBSC-CPV and GaAs control cell were fabricated by adopting a *p* – *i* – *n* cell structure on an *n*-type GaAs (001) substrate using the molecular beam epitaxial method. Details regarding the structure of the solar cell designs are presented in supplementary information S1.

First, we investigated the IB carrier dynamics of the QD-IBSC without light concentration, in order to verify the suitability of samples before integration into the CPV module. To assess the IB dynamics we employed the infrared biased external quantum efficiency (IR-EQE) method at room temperature (RT)^2^,^9^. Since the EQE is the ratio of collected carriers to incident photons at given wavelength hitting the SC, it is a measure of both the absorption and carrier collection. It involves a two-step photo-absorption process at the IB of the QD ensemble due to the IR light illumination. It detects experimentally the difference in the RT-EQE signal between samples measured without IR bias light and those sequentially illuminated by IR light during the test. Due to the very weak RT-IR-EQE signal, the measurement is extremely sensitive to noise. Details regarding the background signal, signal/noise ratio, and the experimental uncertainties are given in supplementary material S2. Fig. 2(a) shows the normal RT-EQE (without IR) for both the GaAs control cell and InAs/GaAs QD-IBSC. Compared to the GaAs control cell, the EQE signal of the InAs/GaAs QD-IBSC beyond 900 nm (the GaAs absorption edge) is clearly visible. These signals were attributed to carriers that were excited from the valence band (VB) to IB states of the QDs and were then thermally excited and collected by the electrode. The RT-IR-EQE results are given in Fig. 2(b). We focus our attention on the region between 920 nm and 1050 nm, where the reliability of the signal was confirmed. Compared to the negligible signal of the GaAs control cell, the InAs/GaAs QD-IBSC exhibits a visible spectral response owing to the IR light illumination. An enhanced EQE signal was detected that can only be attributed to the optical transitions from IB states of the QDs to the higher band above the GaAs conduction band edge^9^. In order to verify further the origin of the enhanced IR-EQE signal in the InAs/GaAs QD-IBSC, a bias voltage was applied to the sample and varied from −0.2 V to +0.2 V. The effect of the bias voltage on the RT-IR-EQE signal is schematically presented in Fig. 2(c). When the sample is under forward bias, the number of carriers drifting across the QD due to the *pn*-junction electric field is reduced. This increases the number of carrier captured within the QD, therefore enhancing the IB-CB absorption under IR illumination. On the other hand, when the sample is under reverse bias, the carrier drift velocity is greatly increased, thus reducing the probability of carriers being captured, and consequently excited by IR photons in the QD region, Fig. 2(c). Experimentally, we observed a similar tendency, wherein the RT-IR-EQE signal increases with increase of the forward bias voltage. Excessive increase of the forward bias voltage tends to reduce the IR-EQE signal as carrier capture becomes dominant and strongly suppresses carrier excitation out of the barrier by IR photons.

It is generally accepted that at room temperature, the majority of carriers are excited out of the QD's potential from the IB by thermal excitation, therefore dramatically reducing the number of carriers participating in two-step photo-absorption. This is the main reason for the weak RT-IR-EQE signal shown in Fig. 2(b). In order to confirm the more pronounced intermediate band features in the InAs/GaAs QD-IBSC samples, we conducted IR-EQE measurements at low temperature (LT-IR-EQE). The LT-IR-EQE experiment was performed using our bespoke equipment, with a basic set-up similar to those reported elsewhere^2^. The wavelength of the filtered IR bias light was longer than 1500 nm and the IR light was chopped at a frequency of 233 Hz. Fig. 3(a) shows a series of representative low temperature EQE results. It is clearly seen here that decreasing the temperature tends to shift the absorption edge of the InAs/GaAs QD-IBSC to a shorter wavelength. We also found that decreasing temperature suppresses carrier collection for both the InAs/GaAs QD-IBSC and GaAs control cell in the range between 700 nm–820 nm. Fig. 3(b) shows LT-IR-EQE results and a dramatically enhanced IR-EQE, of for more than 1%, found for the InAs/GaAs-QDSC at 9K. Since thermal excitation at this temperature can be completely ruled out, the observed increase of 1% in the IR-EQE is solely attributed to the radiative carrier generation. This generation can only originate from two-step photo-absorption involving the IB states, under IR illumination, indicating that the IB is functioning well in our samples.

Having confirmed the carrier dynamics via IB-states for the selected InAs/GaAs QD-IBSC samples, we proceeded with the integration of those cells into the CPV module and investigated the IB-state carrier dynamics under light concentration.

The QD-IBSC-CPV module characterisations were performed at room temperature using HELIOS 3198, a standard CPV measurement system with a flash lamp as a light source. Fig. 4(a) shows the current-voltage curves of the QD-IBSC-CPV and GaAs control modules measured under light concentration of 116 suns. We obtained a conversion efficiency of *η* = 19.2% for the GaAs control module and *η* = 15.3% for the InAs/GaAs QD-IBSC-CPV module. In Fig. 4(b) the measured efficiency *η*(X) is given as a function of the concentration ratio, X. For the GaAs control CPV module, we found that its efficiency reaches a peak plateau in the range X≈140–160 suns and quickly falls off for values of X beyond this. For the QD-IBSC-CPV module the peak plateau is located at around X≈110–130 suns. Theoretically, *η*(X) increases logarithmically with respect to X, as long as the linearity of the short circuit current, *I*_sc_, as a function of X holds^12^. However, since the increase in concentration ratio causes an increase in current, this effect offsets the voltage gained from the light concentration by a potential drop due to the existence of series resistance, *R_s_*, at the electrodes. We have simulated *η*(X) by using the parametric method^13^, in which the “*loss factor*” is given as 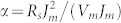, where *J_m_* and *V_m_* are the current and voltage at the maximum operation point. The loss factor represents the fraction of the output power delivered by the solar cell that is lost due to the finite value of *R_s_*. Fitted results shown in Fig. 4(a) were obtained with *R_s_* = 0.05 Ωcm^2^ for both modules. The diode ideality factor was set as 1.0 for the GaAs control module and 1.5 for the QD-IBSC-CPV based module, in order to take into account the existence of an additional recombination path via the IB.

In order to identify the role of the IB in radiative processes, we proceed our analysis with a detailed investigation of the dependence of *I*_sc_ and *V*_oc_ on the concentration ratio, X, in CPV modules. The data shown in Fig. 5 are the normalised *I*_sc_(X)/*I*_sc_(X = 1sun) and *V*_oc_(X)/*V*_oc_(X = 1sun) using results from all four sub-modules. In Fig. 5(a), we clearly noticed a different rate of increase in *I*_sc_(X)/*I*_sc_(X = 1sun) between the GaAs control and the QD-IBSC-CPV module. First, we took an average of *I*_sc_ of all four sub-modules and then performed a linear regression analysis, based on a linear least square fitting algorithm. We found that the rate at which *I*_sc_(X) increases with X, or the slope, *dI*_sc_(X)/*d*X, of the GaAs control module is 0.0061, while the slope for the QD-IBSC-CPV module is 0.0070. As shown in the theoretical analysis below, an increase of *dI*_sc_(X)/*d*X in the QD-IBSC-CPV module, when compared to the GaAs control cell, is one of the decisive signatures of the proper electron dynamics in an IBSC under light concentration.

Results for the open circuit voltage, *V*_oc_(X) vs. X for all four sub-modules are shown in Fig. 5(b). We found that the *V*_oc_ of the QD-IBSC-CPV module is about ~0.25 V lower than in the GaAs control module. The amount of this reduction is consistent with our previous reports^14^,^15^. Lower *V*_oc_(X) in the QD-IBSC-CPV module is mainly due to the fact that recombination via the IB is still prominent. In addition, the VB-CB energy gap in an InAs QD-IBSC is usually smaller than the VB-CB energy band gap of a GaAs cell, which causes greater thermalization of the VB states, i.e., greater dark saturation current. This is therefore also responsible for the reduction of *V*_oc_. Meanwhile, the non-radiative recombination via defects formed at the InAs/GaAs QD hetero-interface is also partly responsible for the observed reduction in *V*_oc_. Fig. 5(b) show results of the linear regression fitting, similar as for *I*_sc_. We conclude that there is a clear increase of *dV*_oc_(X)/*d*X for all four QD-IBSC-CPV modules, when compared to the GaAs control.

In order to gain a better understanding of the experimental results, we performed theoretical calculations to identify carrier dynamics of the QD-IBSC-CPV under light concentration. A schematic model of the IBSC is shown in Fig. 6(a). The current continuity equation for electrons and holes under steady state is given, including IB, as 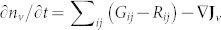, where *v* denotes the type of carriers (*e* or *h*) and 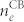, 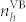, 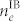 are the electron or hole density of the CB, VB, and IB respectively. *G_ij_* and *R_ij_* (see Fig. 6(a)) are the carrier generation and recombination rates among different bands, *i* and *j*, respectively. In the steady state, which relevant for SC operation, *∂*/*∂t* = 0. We applied the charge conservation rule, 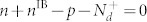, where 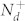 is the pre-filled donor concentration of the IB. In addition, in order to accurately determine the parameter set for the drift-diffusion model, the multiband **k** · **p** method for the electronic structure of the QD arrays, with periodic boundary conditions, was used^16^. From electronic structure model we have obtained information such as the density of states and band gap energies, as well as the respective optical absorption coefficients in the dipole approximation: 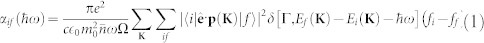
where, *i*, *f* ∈ (VB, IB, CB), **K** is the wave vector of the QD array Brillouin zone, 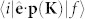 is the polarisation sensitive optical dipole matrix element, Ω = *L_x_* × *L_y_* × *L_z_* is the volume of the primitive cell of the QD array, where *L_z_* = *d_z_* + *h* + *L*_WL_, and *δ* is the spectral line with line broadening Γ^17^. In our electronic structure simulation, we have used experimental structure parameters for the QDs: base length of *b* = 15 nm, height, *h* = 4 nm, and truncation factor *t* = 0.5. Vertical periodicity of the QD array is controlled by the distance between the top of the QD in *n*^th^ layer and the bottom of the wetting layer in the (*n* + 1)^th^ layer growth, and set to *d_z_* = 5 nm. Partly delocalised charge density of IB's states *e*_0_ and *e*_1_ in the CB and *h*_0_ and *h*_1_ in VB are given in Fig. 6(b). Assuming favourable dynamical conditions, that support the formation of quasi-Fermi level separation between CB–IB, VB–IB, and VB–(CB-IB) (where the latter denotes the separation between states in the VB and those in the CB without IB states), the absorption spectra of the QD-IBSC can be decomposed into three different absorption pathways, as shown in Fig. 6(c). It should be noted that, while *α_V_*_,*I*_ and *α_V_*_,*C*_ occure between interband transitions, dominated by the radiative processes and narrow line broadening of, Γ = 5 meV, *α_I_*_,*C*_ occurs due to intraband transitions, which are dominated by much faster nonradiative processes, with much larger and more uncertain line broadening.

Theoretically, for a conventional solar cell under a moderate (several hundred) value of X, *I*_sc_ increases linearly while *V*_oc_ increases logarithmically with respect to X. However, this is not valid for the IBSC even at low concentration ratios. As shown in Fig. 7(a), when compared to the linear variation of *I*_sc_ for the GaAs control cell, the *I*_sc_ of the QD-IBSC-CPV shows a distinct non-linear behaviour. It is apparent that the increase of *dI*_sc_/*d*X for the QD-IBSC-CPV is much larger than that for the GaAs control cell. This confirms our experimental findings shown in Fig. 4(b). Therefore, we conclude that the larger rate at which *I*_sc_ increases with X, observed for the QD-IBSC-CPV module, is a direct consequence of the carrier dynamics via the IB. To examine the linearity of *I*_sc_ in more detail, we normalised *I*_sc_ with concentration ratio X, Fig. 7(b). For the GaAs control cell, such a normalised figure is a constant indicating a linear increase of *dI*_sc_/*d*X. However, in contrast to the GaAs control cell, *I*_sc_(X)/X of the QD-IBSC-CPV shows a logistic-like profile, indicating a strong nonlinear dependence of *I*_sc_(X)/X on X. The difference in behaviour of the QD-IBSC-CPV from the GaAs control cell arises from the complexity of the carrier generation rates associated with the electron occupancy of the IB^18^. As light concentration ratio X increases, the photofilling effects come into play and the generation rate of the IB is greatly enhanced.

The simulation results for *V*_oc_ of QD-IBSC-CPV show a similar tendency to *I*_sc_. The increased rate of *V*_oc_ is much larger for the QD-IBSC-CPV than for the GaAs control cell, especially at low X (below 50–100 suns), Fig. 7(c). The inset in Fig. 7(c) shows that *V*_oc_ of the IBSC is initially lower than that of the GaAs control cell, but reaches a comparable value when X exceeds 1000 suns. The increase of *V*_oc_ with X is attributed to the fact that a larger voltage is required to reduce the large net carrier generation via the IB and to maintain the aforementioned IB continuity.

It is worth mentioning that there has been a general concern regarding the carrier dynamics involving *I*_sc_ enhancement via the IB in the InAs/GaAs QD-IBSC at room temperature (RT), due to the thermal fluctuation^7^,^19^. Our group has reported several successful observations of two–step photon absorption via the IB at RT in samples such as Si doped InAs/GaAs QD-IBSC^9^,^15^. We have found that the suppression of non-radiative recombination loses and the pre-filling of IB due to Si doping played decisive roles in the observation of two–step carrier dynamics at RT. In the current work, InAs/GaAs QD-IBSC was prepared without doping. We attribute the observed carrier dynamics via the IB to the fact that the light concentration has played a similar role to the IB as a pre-filling of the IB in our previous samples^9^. It is known that pre-doping and photo-filling of the IB^20^ play similar roles, which have also been confirmed in our simulation, see Fig. 7(a,b). Meanwhile, it has been reported that the light concentration induced high level of injection can effectively saturate the Shockley-Read-Hall (SRH) type defects, thus suppressing the non-radiative recombination in a multi-quantum well GaAs/AlGaAs solar cell^21^. According to the SRH kinetic statistics, while assuming an *n*-type material with doping concentration, *N_D_*, the capture rate for the minority carrier (hole) can be written as: *R_p_* ∝ *N*^−^Δ*p*, where *N*^−^ is the density of negatively charged defect levels and Δ*p* is the injected carrier concentration. At high injection level, Δ*p*/*N_D_* becomes very large and *N*^−^ → 0, since *R_p_* is approximately constant. Therefore the recombination centres are saturated for minority carriers^22^. In addition, except for the thermal fluctuation effect mentioned above, we have also studied the effect of optical absorption from the IB to the CB on the variation of *I*_sc_(X) as a function of X. The results are summarised in Fig. 7(d). It shows that for IB to CB absorption coefficients as small as 100 cm^−1^, the difference in *dI*_sc_(X)/*d*X vs. X, between the QD-IBSC-CPV module and GaAs control module becomes indistinguishable. However, when the IB to CB absorption coefficient increases to 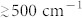, which is below the reported experimental value for the VB to IB absorption coefficient^23^, the difference in *dI*_sc_(X)/*d*X becomes clearly distinctive. Based on our **k** · **p** calculations, a low intraband absorption coefficient value such as 500 cm^−1^ can be obtained by assuming large line broadening, 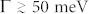. Such a large broadening is fully justified by very fast dynamical intraband nonradiative dynamical processes both experimentally^23^,^24^,^25^ and theoretically^17^ and follows from the Heisenberg relation Γ ∝ ħ/*τ_CB_*_,*IB*_, where *τ_CB_*_,*IB*_ is the electron relaxation time from CB to IB. This indicates that the different *dI*_sc_(X)/*d*X for a QD-IBSC-CPV can be observed even in a system where the IB is relaxed to its discrete energy levels.

In conclusion, we have reported a successful fabrication and characterisation of an InAs/GaAs QD-IBSC-CPV module. In contrast to previous studies, we have demonstrated that our device works in the IB regime and not only as a QD solar cell device. Following the experimental results along with theoretical analysis, we have concluded that: (i) enhanced EQE signal detected in QD-IBSC-CPV when compared to GaAs control cell is due to the optical transitions from the IB, and not the thermal escape; (ii) QD-IBSC-CPV device exhibits an increase of *dI*_sc_/*d*X and *dV*_oc_/*d*X under concentrated light when compared to GaAs control, which is due to the radiative processes involving states in the IB; (iii) theoretical predictions also show increase of *dI*_sc_/*d*X as a function of X for realistic *α_I_*_,*C*_ ~ 500 cm^−1^ and above, when compared to GaAs control, and agrees with experimental observations. The features observed in the behaviour of *I*_sc_(X) vs. X and *V*_oc_(X) vs. X for the QD-IBSC-CPV module are clearly the consequence of the carrier dynamics involving states in the intermediate band. Demonstration of the intermediate-band carrier dynamics in the QD-IBSC-CPV module greatly enhances the feasibility of its practical application with inorganic QD structures and paves the way toward future development of the next generation of solar cells.

## Author Contributions

T.S. and K.Y. performed experiments, simulations, and prepared the manuscript. Y.S., M.O., R.T., H.-F.H. and C.-H.W. performed experiments. C.-T.K. planned the project. S.T. developed the theory and edited the manuscript. Y.O. designed the experiments, edited the manuscript and supervised the project.

## Supplementary Material

Supplementary Information Intermediate-band dynamics of quantum dots solar cell in concentrator photovoltaic modules

## Figures and Tables

**Figure 1:**
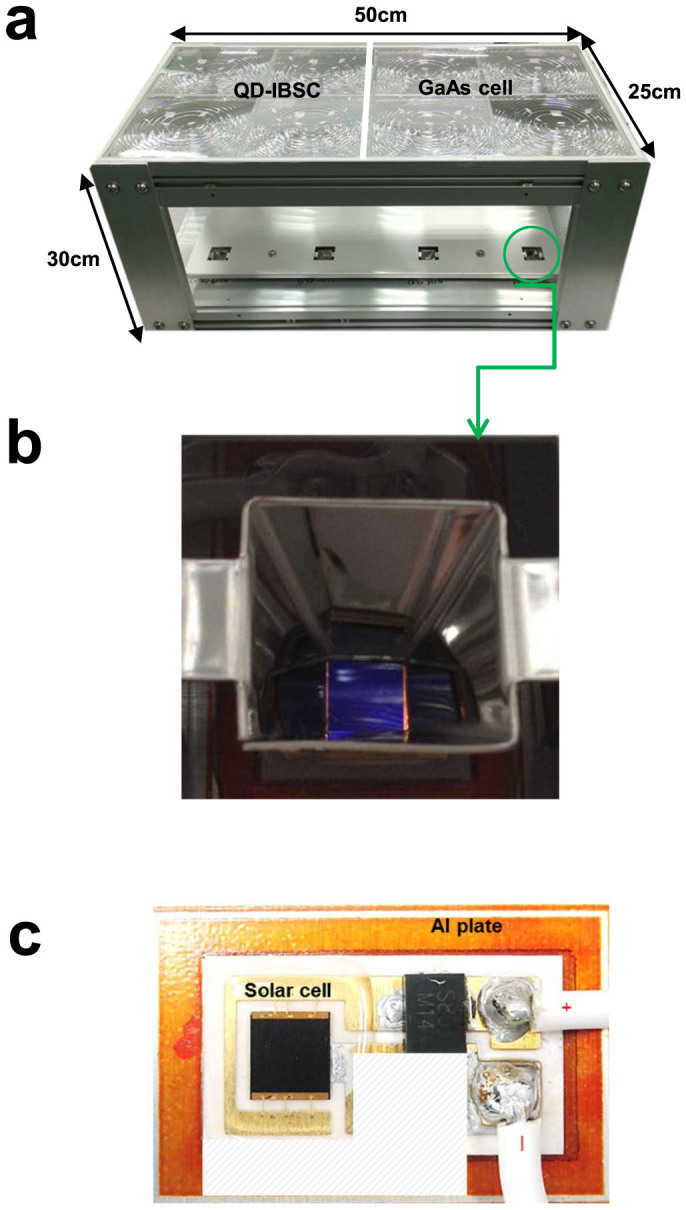
(a) Picture of the fabricated CPV module. (b) A close-up of the secondary optics. The solar cell was covered by a transparent silicon-gel for protection. (c) The submount of the IBSC on aluminium heat sink plate, where a developed plane soldering technique has been employed to maximise the heat dissipation. A blocking diode was also integrated into the circuit to prevent reverse biasing.

**Figure 2:**
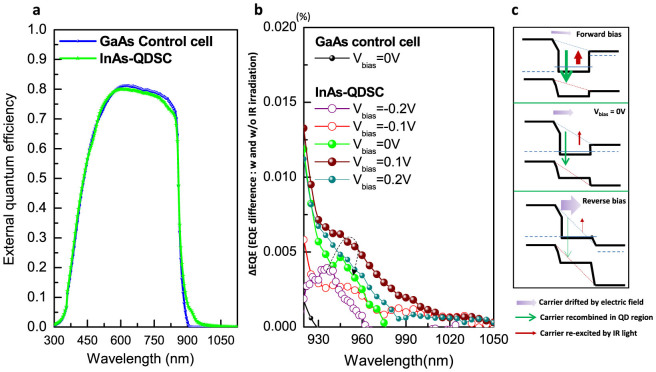
(a) Experimental results of EQE measured at room temperature for both QD-IBSC-CPV and GaAs control cell. (b) EQE difference of solar cell with and without infrared (IR) bias light irradiation as well as the voltage bias dependence. (c) Schematic illustration of bias voltage effect on the intermediate-state carrier dynamics.

**Figure 3:**
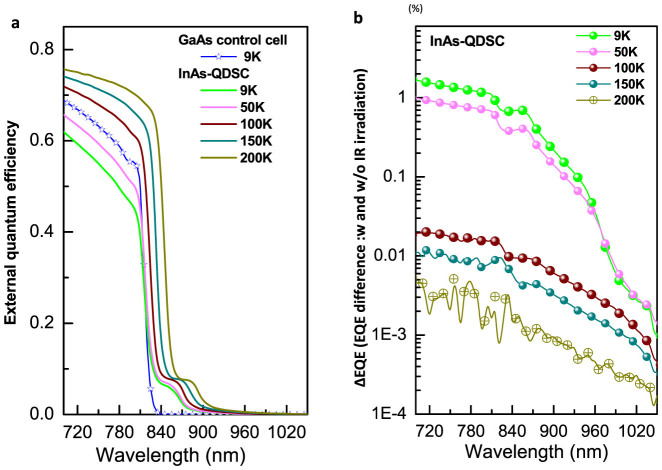
(a) Low temperature experimental results of EQE for both QD-IBSC and GaAs control cell. (b) Low temperature EQE difference of solar cell with and without infrared (IR) bias light irradiation.

**Figure 4:**
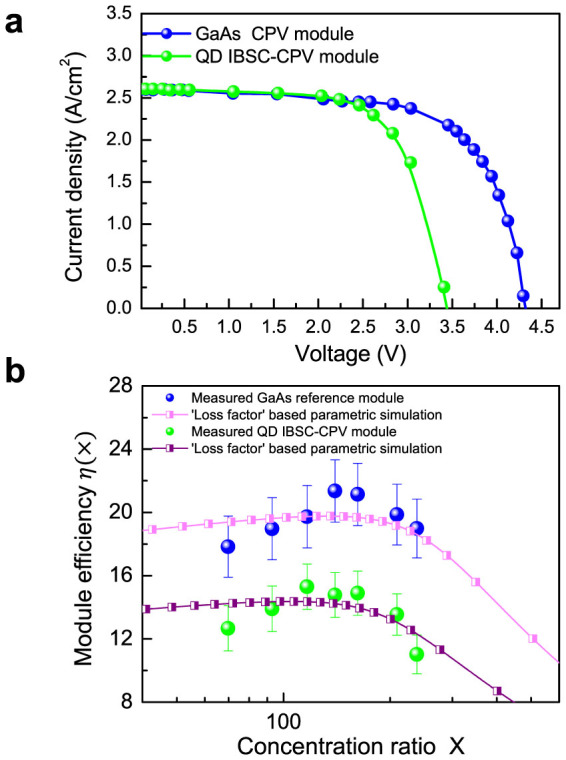
(a) Current-voltage curve of the IBSC-CPV module and GaAs control module. (b) Conversion efficiency *η*(*X*) under different concentration ratio, X. Fitted results, based on “loss factor” parametric method, are also presented.

**Figure 5:**
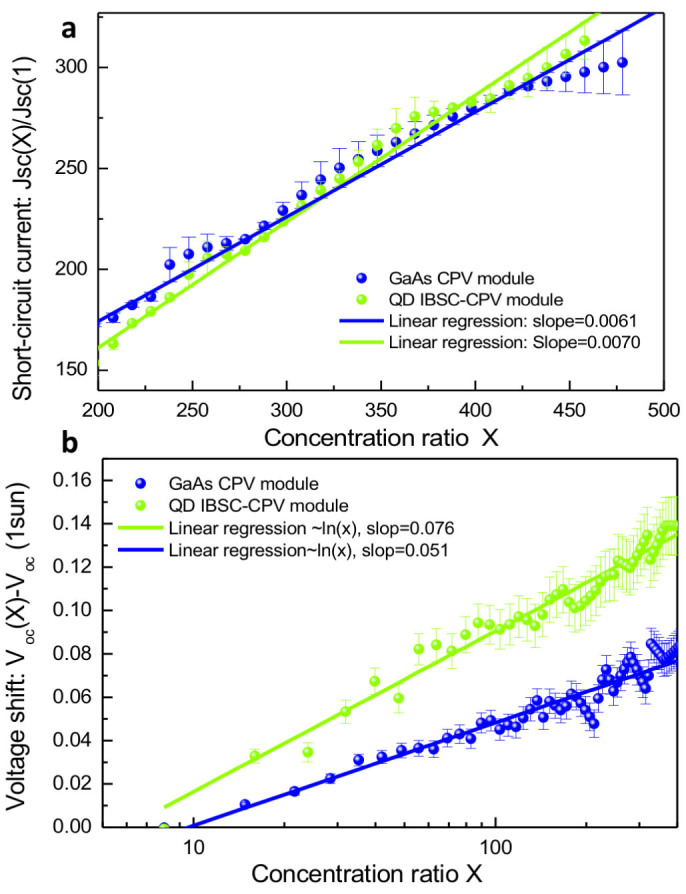
(a) Linear regression fitting of *I*_sc_(*X*) with least square error bars. (b) Linear regression fitting of *V*_oc_(*X*) with least square error bar. Note here that the *V*_oc_(*X*) was shifted by the amount of *V*_oc_(1sun) for better comparison.

**Figure 6:**
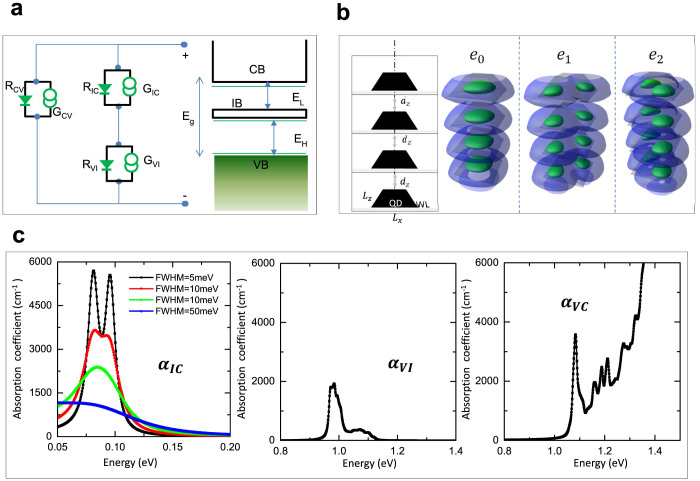
(a) Drift-diffusion model adopted for IBSC. *G_V I_*, *G_IC_*, *G_VC_*, stand for photon generation while *R_VI_*, *R_IC_*, *R_VC_* stand for recombination, where the indices *V*, *I*, and *C* correspond to the valence band, intermediate band and conduction band respectively. Three quasi-Fermi levels (green line) are also given for each band. (b) Geometric information of QD and QD array used in multi-band **kp** calculation. The unit box is represented by three sides of length of *L_x_*, *L_y_* and *L_z_*^26^. (c) Absorption coefficients between IB and CB, VB and IB, and VB and rest of CB.

**Figure 7:**
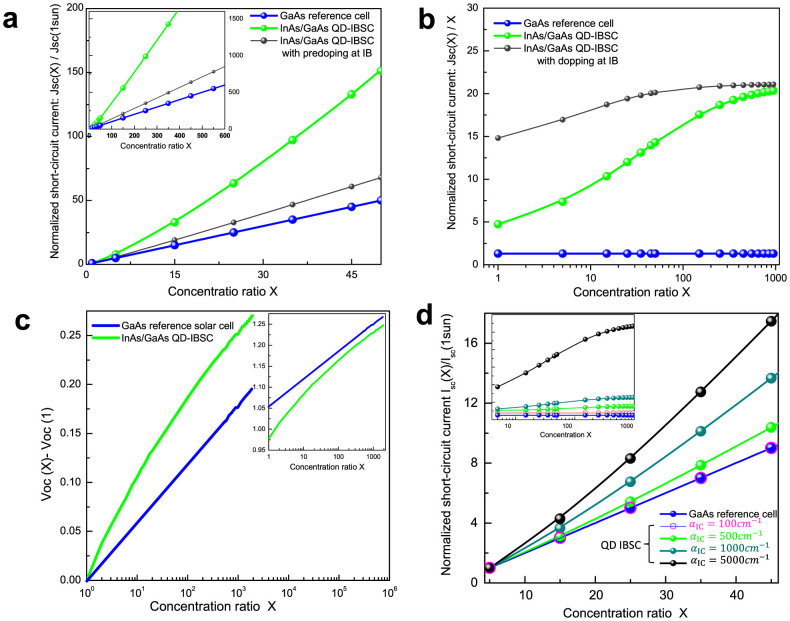
(a) Calculated short circuit current *I*_sc_ for QD-IBSC and GaAs control cell under different concentration ratio. *I*_sc_(*X*) ∝ *X* of IBSC with predoping in IB is also presented (black curve). To compare with experimental data, *I*_sc_(*X*) is normalised by *I*_sc_(1sun). (b) Non-linear feature of *I*_sc_(*X*) of IBSC. Here the *I*_sc_(*X*) is normalised by concentration ratio X and the horizontal axis is set on log scale. (c) Calculated open circuit voltage *V*_oc_ for QD-IBSC and GaAs control cell under different concentration ratio X. *V*_oc_(*X*) is shifted by *V*_oc_(1sun) for better comparison with experimental data. (d) Calculated *I*_sc_(*X*) ∝ *X* for QD-IBSC under different absorption coefficients of the transition from IB to CB.
