# Supplementary material for: Intermediate-band dynamics of quantum dots solar cell in concentrator photovoltaic modules
Source: Sci Rep. 2014 Apr 25;4:4792. doi: 10.1038/srep04792 (PMC3999453; doi:10.1038/srep04792)
Supplement: Supplementary Information — Intermediate-band dynamics of quantum dots solar cell in concentrator photovoltaic modules [file srep04792-s1.pdf]

Supplementary material:

Intermediate-band dynamics of quantum dots solar cell in concentrator photovoltaic modules

Tomah Sogabe[1], Yasushi Shoji[1], Mitsuyoshi Ohba[1], Katsuhisa Yoshida[1], Ryo Tamaki[1], Hwen-Fen Hong[2], Chih-Hung Wu[2], Cherng-Tsong Kuo[2], Stanko Tomić[3], Yoshitaka Okada[1]

[1] Research center for Advanced Science and Technology (RCAST), The University of Tokyo, Tokyo,Japan,
[2]Solar Energy Program, Institute of Nuclear Energy Research (INER), Taoyuan, Taiwan,
[3]Joule Physics Laboratory, School of Computing, Science and Engineering, University of Salford, Manchester, UK

S-1 InAs/GaAs QD growth and device characterization

Epilayers for both GaAs reference solar cells and InAs/GaAs QDs intermediate-band solar cell (IBSC) were fabricated by adopting a *pin* cell structure on n-type GaAs(100) substrate using MBE. For QD-IBSC, the 5 stacked pairs of InAs QDs with 2.19 ML deposition thickness and GaAs spacer layer were inserted into the i-layer and its thickness was tuned in order to suppress or enhance the vertical coupling between QDs. The i-layer was grown at 480℃. The growth rates of InAs QDs layers and GaAs spacer layers were 0.0438 ML/s and 1.0 μm/h, respectively [1]. Fig.1a and 1b show the schematic device model for IBSC and GaAs solar cells. Fig.1c shows the AFM image of the InAs QDs. Fig.1d shows the current –voltage curve for both QD-IBSC and GaAs reference cell. We obtained average efficiency of 19.2% for the GaAs reference cell and 14.3% efficiency for IBSC measured under 1sun.

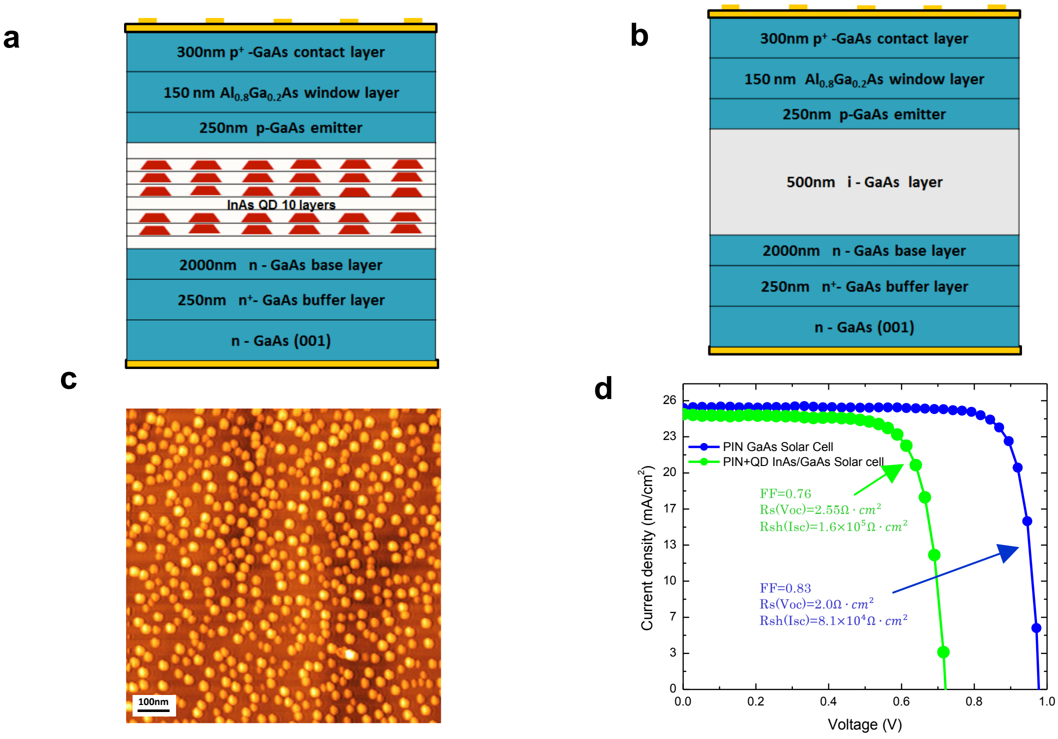

Figure 1 a,b. Schematic of the device configuration for IBSC and GaAs single junction solar cell. **c**. AFM image of the InAs QDs array. **d.** Current-Voltage curve of the IBSC and GaAs solar cell.

S-2 Background signal test for IR-EQE for InAs/GaAs QD-IBSC

In order to examine the measurement system uncertainties such as the fluctuation of sample temperature, ambient temperature, Xe-lamp intensity, we performed background signal test using cyclic RT-EQE measurements for InAs/GaAs QD-IBSC. We have performed five cycles of RT-EQE measurement and obtained RT-EQE difference by subtracting the sequential EQE results. All the repeated RT-EQE tests were performed under IR light illumination. The results are presented in Figure 2 along with the IR-RT-EQE difference between measurement with and without IR irradiation for better comparison. It can be clearly seen here that RT-EQE differences due to system uncertainties are several magnitudes lower than the IR-RT-EQE enhancement due to IR light irradiation. This strongly validates the reliability of our IR-RT-EQE measurement results.

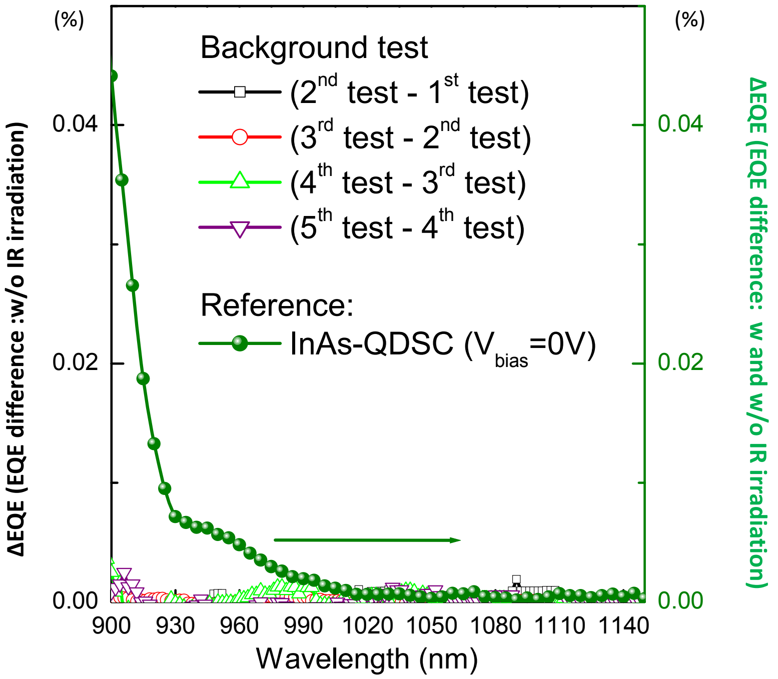

Figure 2 EQE difference from the cyclic EQE measurements under IR light irradiation.

Reference:

[1] Shoji,Y., Narahara, K., Tanaka, H., Kita, T., Akimoto, K. & Okada, Y. “Effect of spacer layer thickness on multi-stacked InGaAs quantum dots grown on GaAs (311)B substrate for application to intermediate band solar cells,” *Journal of Applied Physics* **111**, 074305 (2012).
